# Reviews of Interleukin-37: Functions, Receptors, and Roles in Diseases

**DOI:** 10.1155/2018/3058640

**Published:** 2018-04-01

**Authors:** Hailin Jia, Jing Liu, Bo Han

**Affiliations:** Department of Pediatrics, Shandong Provincial Hospital Affiliated to Shandong University, No. 324 Jingwuweiqi Road, Jinan, Shandong 250021, China

## Abstract

Interleukin-37 (IL-37) is an IL-1 family cytokine discovered in recent years and has 5 different isoforms. As an immunosuppressive factor, IL-37 can suppress excessive immune response. IL-37 plays a role in protecting the body against endotoxin shock, ischemia-reperfusion injury, autoimmune diseases, and cardiovascular diseases. In addition, IL-37 has a potential antitumor effect. IL-37 and its receptors may serve as novel targets for the study, diagnosis, and treatment of immune-related diseases and tumors.

## 1. Introduction

Interleukin-37 (IL-37) was first discovered and identified through computational sequence analysis by Kumar et al. [1] in 2000. It was first named IL-1H4. In 2001, Dunn et al. [2] found that this precursor peptide is the 7th cytokine of IL-1 family, so it was named IL-1F7. Nold et al. [3] found that it could suppress innate immune response and renamed it IL-37. In recent years, IL-37 has been found to play an important regulatory role in the development of a variety of inflammatory diseases, autoimmune diseases, and tumors [3–7]. At present, biological function, signal transduction pathway, and mechanism of immune regulatory functions of IL-37 have not been completely elucidated. This article reviewed the structure, types, function, and disease-related aspects of IL-37.

## 2. Structure, Types, and Processing of IL-37

Human IL-37 gene is located on chromosome 2 with a length of 3.617 kb [4]. IL-37 has a molecular weight of about 17~25 kDa [8]. The structure of IL-37 is similar to that of IL-1 family (IL-1F) and consists of 12*β* tubular lines. The 6 exons encode five isoforms of IL-37 including IL-37a, IL-37b, IL-37c, IL-37d, and IL-37e [4, 8] (Table 1).

IL-37 molecule is an immature precursor peptide, and each isoform is converted from an inactive precursor peptide state to an active state by the cleavage of caspase-1 during expression, and all subtypes regulate each other to form relatively stable state [4]. It is currently believed that caspase-1 cleavage site is located between amino acid residues D20 and E21 expressed by exon 1 [4, 9, 10].

IL-37b (exons 1, 2, 4, 5, and 6) has an intact exon end with the largest molecular weight and has the most complex biological functions. IL-37b transits from an inactive propeptide to an active mature under the action of caspase-1 [4, 11]. Although IL-37a (exons 3, 4, 5, and 6) does not contain exons 1 and 2, its exon 3 encodes a unique N-terminus, and exons 4, 5, and 6 encode IL-1F homologous structure-*β* clover secondary structure. IL-37d (exons 1, 4, 5, and 6) also encodes the 12*β*-strand-containing protein structure, suggesting that these two subtypes also have biological functions [4, 11]. It has been confirmed that IL-37a has an anti-inflammatory effect similar to IL-37b [12].

IL-37c (exons 1, 2, 5, and 6) and IL-37e (exons 1, 5, and 6) do not encode *β*-clover secondary structure due to lack of exon 4, so they may do not have biological functions [4, 11].

IL-37c shares a common N-terminal sequence with IL-37b and IL-37d and competes with IL-37b and IL-37d precursors for the same cleavage enzyme target; whereas IL-37c has no biological function, production of precursor IL-37c is recognized as a mechanism of downregulation of IL-37b and IL-37d [4]. In addition, IL-37e shares the same caspase-1 cleavage site with IL-37b in the region of exon 1, but IL-37e cannot compete with IL-37d due to deletion of exon 2 in the second cleavage site [4]. Therefore, the maturation of IL-37b can be effectively downregulated by IL-37c but only partially downregulated by IL-37e [4].

## 3. Production, Distribution, and Transportation of IL-37

IL-37 is detectable in many human tissues, but the expression level is low in healthy human tissues [3, 4]. IL-37 expression has been detected in human tissues such as liver, lung, thymus, bone marrow, lymph nodes, placenta, testis, uterus, and tumor tissue and in human cell lines such as A431, THP-1, U937, IMTLH, KG-1, HL60, HPBMC, HPT-4, and NHDC [13]. The expression of different isoforms of IL-37 is tissue-specific. IL-37a, b and c are mainly expressed in thymus, bone marrow, lymph nodes, liver, lung, testis, placenta, uterus, colon, NK cells, monocytes, stimulated B cells, and keratinocytes. IL-37d and IL-37e are expressed only in bone marrow and testis, whereas only IL-37a is expressed in the brain, only IL-37b is expressed in the kidney, and only IL-37c is expressed in the heart [2, 4].

IL-37 mRNA is very unstable under normal conditions and can be easily degraded. Stability of IL-37 mRNA is significantly enhanced when it is stimulated by lipopolysaccharide (LPS) or other exogenous stimuli. Stability of IL-37 mRNA is regulated by exon 5 [4, 14]. It is concluded that IL-37 may not play a role in noninflammatory or mild inflammatory state, and its expression is only increased under severe inflammatory conditions to inhibit excessive immune response [4].

IL-37 can be secreted outside to bind to surface receptor to achieve its function. In addition, IL-37 can also be intracellularly secreted. So IL-37 can play an anti-inflammatory effect through both intracellular and extracellular routes [10, 12, 15]. The processing and nuclear translocation of IL-37, as well as the extracellular secretion of mature IL-37, all require the involvement of caspase-1 [10], but secretion of precursor IL-37 does not depend on caspase-1 [10].

Recognizing that IL-37 gene has not been found in mice [4], IL-37-tg mice were widely used as pathological models. IL-37-tg mice were generated using the full-length precursor cDNA of IL-37b isoform driven by the CMV promoter for constitutive expression and IL37-tg mice breed normally and have no obvious phenotype [3].

## 4. Functions of IL-37 and the Mechanisms

As an immunosuppressive factor, IL-37 can suppress innate and adaptive immunity through many ways; furthermore, IL-37 exhibited antitumor effect in several studies (Table 2).

### 4.1. IL-37 in Innate Immunity

Low levels of IL-37 can be detected in peripheral blood mononuclear cells (PBMCs) of healthy individuals [3]. IL-37 expression was significantly upregulated after stimulation by LPS, Pam3CSK4, and TGF-*β*1 [3].

After IL-37 silencing using siRNA against IL-37 (siIL-37) in PBMC, secretions of IL-1*β*, IL-6, IL-12, tumor necrosis factor-*α* (TNF-*α*), granulocyte-macrophage colony stimulating factor (GM-CSF), and granulocyte colony stimulating factor (G-CSF) were significantly upregulated after LPS treatment and showed a dose-dependent manner [3]. The same results were obtained after stimulation using TLR6-TLR2 ligand MALP-2. However, the anti-inflammatory factors IL-10 and IL-1R*α* were not affected [3]. Consistent with this, expression of the proinflammatory cytokines IL-1*β*, TNF-*α*, and IL-6 increased after neutralization of IL-37 produced by PBMCs with anti-IL-37 monoclonal antibodies [12]. In THP-1 cell line and A549 cell line transfected with pIRES-IL-37b plasmid, expression levels of proinflammatory cytokines such as IL-1*α* and IL-1*β* were significantly decreased [3].

Compared with blank control group, TLR ligand-induced release of proinflammatory cytokines from LPS-stimulated mouse macrophage cell line RAW264.7 transfected with pIRES-IL-37b plasmid was significantly inhibited. The inhibitory rates of IL-1*α* and IL-6 were up to 88% and 86%, respectively [3], while the expression of anti-inflammatory cytokines IL-10 and IL-13 was increased [3].

Those findings showed that IL-37 can suppress innate immune response, possibly by reducing the production of proinflammatory cytokines induced by Toll-like receptor (TLR) agonists [3, 8]. IL-1*β*, TNF, IFN-*γ*, IL-18, TGF-*β*, and TLR ligands and inflammatory cytokines can effectively induce the high expression of IL-37, while GM-CSF combined with IL-4 can inhibit its expression [3]. IL-37 can reduce the production of proinflammatory cytokines such as IL-1*α*, IL-1*β*, IL-1Ra, IL-6, IL-8, IL-17, IL-23, TNF-*α*, and IFN-*γ* and chemokines such as MIP-2/CXCL2, CCL12/MCP-5, and BCA-1/CXCL13. It can also inhibit the expression of M-GSF and GM-CSF but increase the production of TGF-*β*1, which is realized as an immunosuppressive factor [3, 8]. It has been reported that IL-37 can induce the expression of nitric oxide (NOS2) in vitro and NOS2 can effectively inhibit the activation of inflammasome and the differentiation of Th17 [16].

Beside the effects on the function of TLRs, IL-37 can also suppress innate immune response by inhibiting the activation of the inflammasome of the NOD-like receptor family Pyrin Domain-Containing 3 (NLRP3) [3, 16], and NLRP3 plays a key role in various inflammation-related signal transduction pathways [17, 18]. In lung aspergillosis mouse model, compared with untreated aspergillosis mice, expression of NLRP3 mRNA was significantly decreased in mice intraperitoneally injected with IL-37, while levels of myeloperoxidase (Mpo), CxCl2, IL-1*β*, IL-17A, and IFN-*γ* were significantly reduced [16]. IL-37 attenuates the production of proinflammatory cytokines and recruitment of neutrophils to the lungs by inhibiting the activity of NLRP3 inflammasome [16]. The experiment further confirmed that IL-37 lost its regulatory effects on neutrophil recruitment and expression of IL-1*β*, MPO, and CXCL2 in NLRP3-deficient mice [16]. Bulau et al. [10] also confirmed that the inhibitory effect of IL-37 on expression of IL-6 and IL-1*β* was significantly weakened in bone marrow macrophages of NLRP3 or associated apoptosis-associated protein-containing carboxyserine caspase recruitment domain- (ASC-) deficient mice.

### 4.2. IL-37 in Adaptive Immunity

IL-37 is a natural inhibitor of innate immunity, whereas adaptive immunity relies on innate immunity. Moretti et al. [16] have found that IL-37 can significantly inhibit the activation of Th2/Th17 cells in mice with allergic bronchopulmonary aspergillosis, indicating that IL-37 may also affect adaptive immunity.

Dendritic cells (DCs) are important antigen-presenting cells in the body. DCs accept and integrate signals from a variety of cells and transmit the signals to a variety of immune cells, so as to play an important role in the initiation and development of adaptive immune responses [19]. DCs have the ability to induce both immune responses and immune tolerance [19]. Studies have shown that IL-37 can induce DCs to acquire immune tolerance and thus inhibit adaptive immune response [20]. In skin contact hypersensitivity (CHS) mouse model, swelling of the local skin of the allergen-vaccinated IL-37tg mice was significantly reduced compared to the wild-type mice. Antigenic response was also significantly weakened in wild-type mice after treatment with hapten sensitized DCs [20]. In vitro experiments showed that expression of MHC II and costimulatory molecules CD40 on the surface of DCs from IL-37tg mice was significantly decreased, their ability to activate naive T cells and antigen-specific T cells decreased, but their ability to induce differentiation of Treg cells was obviously enhanced [20]. Immunohistochemical results showed that the number of CD8+ T cells was significantly reduced, while the number of Treg cells was significantly increased [20]. IL-37 can delay the process of antigen presentation by downregulating the activity of DCs. IL-37 can also affect Th cell balance, thereby attenuating T cell-mediated inflammation [3, 20].

### 4.3. Antitumor Effect of IL-37

In addition to its immunosuppressive effects, antitumor effect of IL-37 has been more and more studied [7, 13, 21]. Gao et al. [13] constructed a mice MCA205 fibrosarcoma model and injected recombinant adenovirus IL-37b (adenovirus-mediated gene transfer, AdIL-37b) directly into the tumor. As a result, a single injection of AdIL-37b significantly inhibited tumor growth, and tumor growth was inhibited completely after multiple injections. B6.Cg-Foxn1^nu^ nude mice and B6.CB17-Prkdc^scid^ /SzJ SCID mice do not have functional T cells and B cells. After injection of AdIL-37b into MCA205 fibrosarcoma of those mice, no suppressed tumor growth was observed. IL-37b also showed no antitumor effects on mice deficient in IFN-*γ*, indicating that IFN-*γ*, functional T cells, and B cells play a key role in the antitumor activity of IL-37 [13]. IL-37b also showed no antitumor effects in IL-12 p40/40 knockout mice. However, the antitumor effect of IL-37b was not reduced in NKT knockout mice, indicating that IL-37 does not achieve its antitumor effect through NKT cells, but the involvement of IL-12 is needed [13]. IL-12 mediates antitumor effects mainly through perforin and the function of IL-18 mainly rely on FasL [22]. After AdIL-37b was injected into MCA205 fibroblast of B6Smm.C3H-Fasl^gld^-FasL ligand-deficient mice, tumor growth was not inhibited, indicating that IL-37b has similar antitumor effects to IL-12 and IL-18, so it can be speculated that IL-37b may be an important mediator of innate immunity and adaptive immunity [13].

In hepatocellular carcinoma (HCC), patients with high expression of IL-37 in tumor tissue have better overall survival rate and disease-free survival rate [21]. In tumor tissues, expression of IL-37 was positively correlated with the density of CD57+ natural killer (NK) cells. In vitro experiments also demonstrated that IL-37 has the ability to recruit CD57+ NK cells [21]. Subsequently, it was confirmed in mouse hepatocellular carcinoma model that IL-37 has the effect of inhibiting tumor growth, possibly by increasing the recruitment of CD57+ NK cells to kill cancer cells [21]. However, the antitumor effect of IL-37 has been proved to have no obvious relationship with CD3+ and CD8+ T cells. This is not consistent with the finding of Gao et al. [13]. This may be due to the different pathogenesis of different tumors and different pathways involved in the antitumor functions of IL-37.

IL-37 also showed antitumor effects in mouse model of non-small-cell lung cancer [23]. Compared with control group, transfected mice stably expressing IL-37 showed significantly reduced tumor growth rate, microvessel density, and expression levels of VGEF and CD34, and IL-37 treatment also significantly inhibited the growth and angiogenesis of human umbilical vein endothelial cells (HUVECs) [23]. It is speculated that IL-37 may inhibit tumor angiogenesis and thus play a role in tumor suppression [23]. However, IL-37 did not show significant antitumor effect in vitro experiments. It is speculated that IL-37 may rely on different pathways in different tumors to achieve its function, for example, by enhancing the function of effector T or altering the tumor microenvironment to achieve antitumor effects.

IL-37 also showed inhibitory effect on tumor cells in renal cell carcinoma [24] and cervical cancer [25]. Those studies showed that IL-37 can inhibit tumor cell migration, proliferation, and induce cell apoptosis possibly by inhibiting STAT3 expression and phosphorylation [24, 25].

### 4.4. IL-37 Related Receptors and Signaling Pathways

#### 4.4.1. IL-37b and Smad3

After human epithelial cell line A549 was transfected with FLAG labeled IL-37b, IL-37b and phospho-Smad3 (mothers against decapentaplegic related protein) were colocalized in perinuclear regions and cytoplasm. Anti-FLAG coimmunoprecipitation further confirmed the interaction between IL-37b and Smad3 [3]. IL-37 can enter the nucleus to form functional complexes with Smad3, thus affecting gene transcription and inhibiting Toll-like receptor-induced expression of proinflammatory cytokines IL-1*β*, IL-16, and IFN-*γ* [3, 4]. Anti-inflammatory properties of IL-37 were found to be markedly reduced in mouse RAW264.7 cell line and human THP-1 cell line after the use of Smad3-specific inhibitor or silencing of endogenous Smad3 using targeted siRNA [3]. Knockdown of Smad3 in the lungs of IL-37 transgenic mice with siRNA also markedly diminished the anti-inflammatory effects of IL-37 [3]. These results indicate that Smad3 is essential for the anti-inflammatory effects of IL-37.

#### 4.4.2. IL-37b Inhibits STATs and p38MAPK

Phosphorylated STAT1-4 is a key player in the signaling transduction of proinflammatory cytokines. It is an important signaling molecule in signal transduction of proinflammatory cytokines IL-6, IL-12, and IFN-*γ* [26], while phosphorylated p38MAPK is centrally located in a variety of proinflammatory signaling pathways [27]. IL-37 and Smad3 can form a functional complex to inhibit the activity of many kinds of STATs and reduce the phosphorylation of p38MAPK, ERK1/2, and JNK, as well as the expression of some kinases involved in inflammatory signaling pathways such as focal adhesion kinase (FAK), proline-rich tyrosine kinase 2 (Pyk2), and paxillin [3, 8, 12, 28]. Xie et al. [29] have shown that IL-37 can significantly inhibit NF-*κ*B signaling pathway. Nold-Petry et al. [30] found that the binding of IL-37 to IL-1R8 inhibited the expression of kinase Fyn, TAK1, NF-*κ*B, and MAPK through inhibiting signaling molecules Mer, PTEN, STAT3, and p62.

#### 4.4.3. IL-37b and IL-18 and Their Receptors

IL-18 is a proinflammatory cytokine that can induce the proliferation and differentiation of T cells, promote and maintain the secretion of IL-17 by Th17 cells, and promote Th17 cells-based cellular immune response [31, 32]. IL-37 has two conserved amino acid residues (Glu-35 and Lys-124) that are structurally similar to the two conserved residues of IL-18 (Glu-35 and Lys-89) [9, 33–35], indicating that IL-37 and IL-18 may have the same receptor. Studies have shown that IL-37b can bind to IL-18R*α*, but it is not an agonist or antagonist of IL-18. Chemically cross-linking shows that IL-37b cannot recruit IL-18R*β* to form a functional trimer [9, 33], and the binding affinity of IL-37 to IL-18R*α* is only 1/50 that of IL-18, so it cannot competitively bind to IL-18R*α* with IL-18 [9]. However, IL-37 can bind to IL-18R*α* to form a complex with IL-18BP, a natural antagonist of IL-18, so as to enhance the inhibitory effect of IL-18BP on IL-18, thereby reducing the activity of IL-18 and the levels of Th1 cells, NK cells and IFN-*γ* [9, 16, 33, 36]. IL-37b can bind to IL-18BP to form a complex with IL-18R*β*, which can reduce the formation of IL-18R*α*/*β* complex and thus inhibit the signal transduction pathway of IL-18 [33].

IL-37 also need to bind to single immunoglobulin IL-1 receptor related protein (SIGIRR) to exert an anti-inflammatory effects [12]. SIGGRR is a newly discovered member of the Toll-like receptor/Interleukin-1 receptor (TIR) superfamily. SIGGRR is also known as TLR-IL-1R8 (TIR8) and can regulate Th2 immune response [37]. It has been demonstrated that IL-37 cannot exert its anti-inflammatory effects in bone marrow DCs (BMDCs) of SIGGRR/IL-R8 deficient mice [12]. Experiments by Moretti et al. [16] on mice pulmonary aspergillosis model have demonstrated that IL-37 fails to exert its anti-inflammatory effect by inhibiting the activation of the NALP3 inflammasome in TIR-8/SIGIRR-deficient mice (Tir8 −/− mice). Nold et al. [38] found that IL-37 can exert its anti-inflammatory effect in combination with IL-18R*α*, while anti-inflammatory effect of IL-37 disappeared in SIGIRR knockout mice (IL-37tg-SIGIRR-KO mice). Nold-Petry et al. [30] also found that IL-1R8 and IL-18R*α* were required for IL-37 to exert its anti-inflammatory effects both in vivo and in vitro.

## 5. IL-37 in Disease

### 5.1. IL-37 in Infectious Diseases

Endotoxin LPS from Gram-negative bacteria can induce the excessive production of inflammatory mediators such as TNF-*α*, IL-1, IL-6, IL-8, oxygen free radicals, and histamine, which in turn lead to septic shock. Nold et al. [3] found that, compared with wild-type mice, alleviated inflammatory response, weakened LPS-induced hypothermia, metabolic acidosis, dehydration, hyperkalemia symptoms and liver injury and other endotoxic shock performance, and increased respiratory compensation were observed in mice transplanted with human IL-37 precursor gene (transgenic mice for human IL-37 precursor, hIL-37tg) after intraperitoneal injections of LPS. Compared with wild mice, 16 proinflammatory cytokines and 2 chemokines in serum were reduced by more than 33%. Improved toxemia manifestations were found in IL-37tg homozygous mice compared to those in heterozygous mice. Percentage of CD86 and MHC II molecules double positive cells (average 47%) was significantly lower in those treated mice than in wild-type mice (average 73%), and the DC activity was inhibited in those mice [3]. This indicates that IL-37 can inhibit the occurrence of septic shock by inhibiting the expression of proinflammatory cytokines.

A clinical study on chronic hepatitis B showed that serum levels of IL-37 in chronic hepatitis B patients with high viral load were significantly higher than those in normal population, and serum levels of IL-37 were increased in hepatitis B e antigen- (HBeAg-) positive chronic type B patients and were positively correlated with the virus concentration and ALT levels [39]. It is speculated that IL-37 plays an important role in the immune tolerance of chronic HBV infection.

In HIV-infected patients, IL-37 mRNA expression was significantly increased in PBMCs compared with noninfected patients, and the steady-state level of IL-37 mRNA in PBMCs was positively correlated with viral load [40]. A study carried out by Chattergoon et al. [41] showed that IL-37 inhibited the replication of HIV, indicating the potential role of IL-37 in the treatment of HIV infection.

After intraperitoneal injection of IL-37 into mouse model of aspergillus infection, Moretti et al. [16] found that IL-37 effectively reduced the activation of NLRP3 inflammasome, decreased the expression levels of inflammatory factors and chemotaxis factors, and reduced neutrophil infiltration, lung injury, and degree of cystic fibrosis. However, in* C*.* albicans*-induced animal model of diffuse candidiasis, hIL-37tg mice showed more pronounced susceptibility to* C*.* albicans* and the mortality rate (all deaths within 10 days) was significantly higher than that of the control group [42].

Those studies have shown that IL-37 can inhibit excessive immune response and protect the body from endotoxic shock and infectious diseases. But IL-37 also may lead to increased infection and mortality in infection caused by some pathogens possibly due to its inhibitory effects on immune response (Table 3).

### 5.2. IL-37 in Ischemia-Reperfusion Injury

Ischemia-reperfusion (I/R) injury refers to the increased tissue and organ dysfunction and structural damage caused by reperfusion after a period of tissue ischemia [43]. And IL-37 may play a protective factor in I/R injury (Table 4).

A study by Sakai et al. [44] found that, compared with untreated I/R liver injury mice, treatment of I/R-induced liver injury mice with recombinant human IL-37 protein inhibited neutrophil recruitment, decreased levels of injury, and reduced the serum levels of TNF-*α*, MIP-2, and liver reactive oxygen free radicals. Direct application of recombinant IL-37 protein on LPS-stimulated hepatocytes and Kupffer cells inhibited TNF-*α*-induced neutrophils activation, reduced liver cell death, and increased expression level of Bcl-2 [44], and Bcl-2 is an important protective factor against I/R liver injury [45, 46]. In addition, the activation of p38MAPK and JNK signaling pathway is an important mechanism of I/R liver injury [47, 48], and IL-37 may inhibit the activation of p38MAPK and JNK signaling pathways [3], thereby reducing the release of inflammatory cytokines and improving liver damage.

Wu et al. [49] confirmed that administration of recombinant human IL-37 in myocardial ischemia-reperfusion mice could effectively reduce myocardial ischemia-reperfusion injury. Myocardial infarct size and troponin T level were significantly decreased compared with control group, and cardiac function was significantly improved. IL-37 can effectively reduce the expression of proinflammatory cytokines and chemokines and reduce neutrophil infiltration, as well as myocardial cell apoptosis and production of reactive oxygen species (ROS) [49]. IL-37 effectively inhibits the expression of TLR-4 and the activation of NF-*κ*B but increases the level of anti-inflammatory cytokine IL-10 [49]. In vitro experiments also confirmed the protective effect of IL-37 on myocardial ischemia-reperfusion injury [49]. The study also found that protective effect of IL-37 on myocardial ischemia-reperfusion injury was significantly reduced when IL-10 signaling pathway was blocked by a specific anti-IL10R mAb, indicating that the protective function of IL- 37 at least partially depends on the involvement of IL-10 [49]. Yang et al. [50] further confirmed the protective effect of IL-37 on renal ischemia-reperfusion injury in mice.

### 5.3. IL-37 in Autoimmune Diseases

Nold et al. [3] found that IL-37 was excessively expressed in the synovial tissue of patients with active rheumatoid arthritis. Following up studies have shown that IL-37 is closely related to inflammatory diseases and autoimmune diseases [5, 6] (Table 5).

#### 5.3.1. IL-37 in Inflammatory Bowel Disease

Inflammatory bowel diseases (IBD) are idiopathic inflammatory bowel diseases that can affect ileum, rectum, and colon. IBD include ulcerative colitis (UC) and Crohn's disease (CD). Imbalance of immunomodulatory processes, multiple immune cells, and proinflammatory cytokines were involved in the pathogenesis of IBD [51, 52].

McNamee et al. [53] established a transgenic mouse (hIL-37tg mice) expressing human IL-37 and constructed a colitis model using dextran sulfate sodium (DSS). Clinical disease score of IL-37 transgenic mice was 50% lower than that of wild-type mice, and the histological scores of IL-37 transgenic mice were also significantly lower than those of wild-type mice. Expression levels of IL-1*β* and TNF-*α* in colonic tissue of IL-37 transgenic mice were significantly decreased compared with wild-type mice, but expression level of anti-inflammatory cytokine IL-10 was significantly increased [53]. IL-10 showed no significant effects on the function of IL-37, indicating that IL-10 may be not a key player in anti-inflammatory function of IL-37 in a colitis model [53]. The same protective effect was observed in wild-type mice after hIL-37tg bone marrow transplantation, indicating that IL-37b, which is derived from bone marrow, is sufficient for anti-inflammatory effects [53].

Imaeda et al. [54] found that IL-37b was almost not expressed in normal colon tissue and highly expressed in affected colon in IBD patients, and the expression level was positively correlated with the degree of disease. In vitro experiments showed that IL-37b inhibited the activation of T cells and DCs, thereby reducing intestinal inflammation [54]. A study of childhood IBD showed that expression level of IL-37 in patient's colon was positively correlated with the histopathological score of UC or CD [55]. Li et al. [56] found that expression level of IL-37 in colonic mucosa of UC and CD patients was higher than that of healthy controls, while level of IL-37 in serum was lower than that of normal controls, and level of IL-37 in serum was negatively correlated with Mayo score of UC patients. Above studies showed that IL-37 was involved in the development of IBD and had a protective effect on the body.

#### 5.3.2. IL-37 in Systemic Lupus Erythematosus

Systemic lupus erythematosus (SLE) is an autoimmune disease with the activation of T lymphocytes and B lymphocytes and appearance of autoantibodies against autoantigens during the onset of disease [57]. Song et al. [58] found that serum level of IL-37 was significantly higher in patients with SLE than in healthy controls, and IL-37 level decreased after treatment with glucocorticoid, and IL-37 level was positively correlated with IL-18, IL-18BP, IFN-*γ*, IL-6, and SLEDAI score before and after treatment [58]. Ye et al. [59] found that IL-37 mRNA in PBMC and serum IL-37 of SLE patients were significantly higher than those in healthy controls. IL-37 was associated with the activity of SLE disease, and levels of IL-37 were higher in active patients than in inactive patients. Serum IL-37 levels in patients with renal damage were significantly higher than those without renal damage. There was no significant difference in serum IL-37 level between healthy control and patients without renal damage, suggesting that IL-37 is closely related to renal damage in SLE [59]. In vitro experiments showed that recombinant IL-37 can inhibit the expression of TNF-*α*, IL-6, and IL-1*β* in PBMCs from SLE patients [59]. It is speculated that IL-37 may play a protective role in the development of SLE.

#### 5.3.3. IL-37 in Rheumatoid Arthritis

Rheumatoid arthritis (RA) is a chronic systemic inflammation and autoimmune disease mainly characterized by joint diseases. A variety of cytokines are involved in the pathogenesis of rheumatoid arthritis. Dysfunction of immune regulation is the leading cause of rheumatoid arthritis [60].

Zhao et al. [61] found that serum levels of IL-37 in patients with RA were significantly higher than those in healthy controls. After DMARD treatment, serum IL-37 levels were decreased in drug susceptible patients and levels of IL-37 are closely related to proinflammatory cytokines (IL-17A and TNF-*α*) and RA activity (CRP, DAS28 score). These results are consistent with the findings of Xia et al. [62] and Yang et al. [63]. Serum levels of IL-37 in RA patients were closely related to disease activity (CEP, ESR, and DAS28 score). In vitro experiments showed that IL-37 significantly decreased the expression of IL-17, IL-1*β*, and IL-6 and inhibited the proliferation of Th17 lymphocytes. However, IL-37 had no significant effect on the differentiation of Th17 cells [64]. Treatment in collagen-induced arthritis (CIA) model of mice with Adil-37 resulted in a significant reduction in the incidence of disease and significantly improved disease conditions including synovial hyperplasia, angiogenesis, cartilage damage, and bone erosion in the knee joint. Expression levels of IL-17, IL-1b, and IL-6 were also decreased after treatment [64]. However, a survey on Han population found that the SNP (rs3811047) of IL-37 was associated with clinical manifestations of patients with RA [65]. The number of joint swollenness, joint swelling index, degree of resting pain, and health survey evaluation index in RA patients with AA or AG genotype were lower than those in GG genotype patients, indicating that the A allele of IL-37 gene (rs3811047) may be a protective factor for the activity of RA disease [65].

#### 5.3.4. IL-37 in Psoriasis

Psoriasis is an autoimmune-mediated chronic inflammatory skin disorder and abnormal expression of inflammatory mediators plays an important role in the pathogenesis of this disease [66, 67]. Teng et al. [68] constructed a human keratinocyte cell line (HaCaT) stably expressing IL-37 and found that IL-37 significantly decreased the inflammatory reaction induced by M5 (10 ng/ml TNF-*α*, IL-17A, IL-22, IL-1*α*, and oncostatin-M), compared with the control group, expression of CXCL8, IL-6, and S100A7 was significantly inhibited. Further studies found that, compared with control group, improved local epidermal hyperplasia and ulceration, reduced epithelial hyperplasia, vasodilatation and inflammatory cell infiltration, and reduced symptom score of psoriasis were observed in K14-VEGF transgenic mice (K14-VEGF-Tg mice) injected with IL-37 plasmid [68]. Expression of IFN-*γ* in psoriatic plaques was also significantly reduced in this group of mice [68]. Immunohistochemistry revealed abundant IL-37 expression in human psoriatic plaques, and IL-37 is mainly expressed in memory T cells (TEMs) and macrophages, and expression level was relatively low in epithelial cells, and its expression levels were higher in areas with severe lesions than in areas with lighter lesions [68]. Thus, IL-37 may be an important immunoregulatory factor in the development of psoriasis and exert its effects by inhibiting the expression of key proinflammatory cytokines.

#### 5.3.5. IL-37 in Asthma

Asthma is a chronic inflammatory disease of the airway. A variety of immunocytes and cellular components are related to the development of asthma, and imbalance in immunomodulation is involved in the pathogenesis of this disease [69]. Lunding et al. [70] found that the expression of IL-37 in PBMC of children with allergic asthma was significantly lower than that of healthy controls. In ovalbumin- (OVA-) induced mouse acute asthma model, use of IL-37 nasal inhalation therapy effectively alleviated allergic airway inflammation, decreased the expression levels of a variety of proinflammatory cytokines, and reduced mucus production and airway hyperresponsiveness, indicating that IL-37 can relieve allergic asthma mediated by Th2 cells [70]. However, IL-37 did not show the same effect on IL-18R*α* and SIGIRR-deficient mice, suggesting that IL-37 needs to bind to IL-18R*α* and SIGIRR to exert anti-inflammatory effects [70].

A study on childhood asthma showed that levels of IL-37 in serum and expression level of IL-37 mRNA in induced sputum (IP) in asthmatic children were lower than those in the normal controls and were related to the severity of the disease [71]. Compared with healthy control, IP from asthmatic children showed higher levels of IL-1*β*, IL-6, and TNF-*α* after LPS stimulation, while rIL-37 significantly inhibited the production of IL-1*β*, IL-6, and TNF-*α*, and rIL-37 significantly inhibited the formation of Th17 from CD4+ T cells [71]. It is speculated that IL-37 as an anti-inflammatory may play a critical role in the development of asthma.

#### 5.3.6. IL-37 in Guillain-Barre Syndrome, Ankylosing Spondylitis, and Graves' Disease

IL-37 level was significantly higher in patients with Guillain-Barre syndrome (GBS) [72], ankylosing spondylitis (AS) [73], and Graves' disease (GD) [74] than in healthy controls, and the expression level of IL-37 was closely related to disease activity. Serum levels of IL-37, IL-17A, IFN-*γ*, TNF-*α*, and cerebrospinal fluids IL-37 and IL-17A were significantly increased in the acute phase of GBS and levels of IL-37 and IL-17A in cerebrospinal fluid and serum level of TNF-*α* were positively correlated with the grade of paralysis [72]. Serum levels of IL-37 were positively correlated with levels of IL-6, IL-17, and TNF-*α* in patients with GD and AS, and in vitro experiments confirmed that IL-37 can inhibit secretion of proinflammatory factors by PBMCs [73, 74]. Studies have confirmed that single nucleotide polymorphism of IL-37 gene is associated with AS susceptibility in Han population, and this susceptibility and HLA-B27 may be two independent risk factors for AS [75].

### 5.4. IL-37 in Cancer

Thus far, more and more studies have shown that IL-37 has antitumor effects, so in this portion of the review, the focus will be on the antitumor effects of IL-37 (Table 6).

Gao et al. [13] constructed a mice MCA205 fibrosarcoma model, and AdIL-37b was injected directly into the tumor. Results showed that single injection of AdIL-37b significantly inhibited tumor growth, while tumor growth was completely inhibited after multiple injections, and the antitumor effect of IL-37b required the participation of IFN-*γ*, IL-12, and functional T cells and B cells [13].

Zhao et al. [21] used immunohistochemical methods to analyze clinical samples derived from 163 patients with HCCs. Compared with control group, expression level of IL-37 in HCC group was decreased and inversely proportional to tumor size. This study showed that patients with high expression level of IL-37 in HCC tumor tissue had better overall survival and disease-free survival, and the low expression of IL-37 in tumor tissue was an independent risk factor for poor prognosis [21]. In HCC model of mice overexpressing IL-37, delayed tumor growth was observed and more NK cells were recruited to tumor tissue [21]. This suggests that IL-37 may serve as not only valuable biomarker for prognosis, but also a new target for the treatment of HCC.

IL-37 also showed antitumor effects in mouse non-small-cell lung cancer model [23]. Compared with control group, tumor growth was slower in mice expressing IL-37, possibly related to its inhibitory effects on proinflammatory response and tumor angiogenesis [23].

IL-37 has also been shown to inhibit the growth of tumor cells in renal cell carcinoma [24], cervical cancer [25], and oral squamous cell carcinoma [76], but all experiments were performed in vitro. So the role of IL-37 still needed to be studied in vivo. Pathogenesis of different tumors is various, and IL-37 may play different roles in different tumors. IL-37 may change tumor microenvironment in vivo, so as to play its antitumor effect.

### 5.5. IL-37 in Cardiovascular Disease

A recent epidemiological survey on cardiovascular diseases in Europe showed that cardiovascular disease remained the first cause of death and the incidence of this disease showed an increasing trend [77]. Cardiovascular diseases such as atherosclerosis and acute coronary syndrome are closely related to the development of immune system [78–80].

Chai et al. [81] established an atherosclerosis model using apoE-deficient diabetic mice. They found that, compared with control group, mice with intraperitoneal injection of IL-37 showed significantly reduced arterial calcification area, atherosclerotic plaque size, and lower plaque vulnerability scores in the root of aorta. Treatment with IL-37 elevated serum osteoprotegerin (OPG) concentrations, decreased expression levels of ALP, BMP-2, TNF-*α*, and IL-18, and increased the expression levels of anti-inflammatory factor IL-10. Compared with mice only treated with IL-37, vascular calcification area, atherosclerotic plaque size, and plaque vulnerability scores were increased in mice treated with both IL-37 and anti-OPG antibody, indicating that the function of IL-37 in atherosclerotic mice depends or at least partially depends on OPG [81].

A study on acute coronary syndromes (ACS) showed that serum levels of IL-37, IL-18, and IL-18BP were significantly increased in ACS patients compared to patients with stable angina pectoris (SAP) and healthy controls [82]. Levels of IL-37, IL-18, and IL-18BP were positively correlated with C-reactive protein (CRP), N-terminal probrain natriuretic peptide (NT-proBNP), and left ventricular end-diastolic dimension (LVEDD) and were negatively correlated with left ventricular ejection fraction (LVEF), but all those three factors showed no correlation with the degree of coronary stenosis [82]. Wang et al. [83] found that levels of IL-37, IL-18, and IL-18BP in blood of patients with acute ST-segment elevation myocardial infarction (ASTEMI) were significantly lower than those in healthy control at 12 h, 24 h, and 48 h after percutaneous coronary intervention (PCI). Immunoblotting showed that levels of IL-37 and ICAM-1 expressed by leukocytes were the highest at 12 h and then reduced at 48 h [83]. A study on mouse model of acute myocardial infarction showed that IL-37 inhibited the expression of myeloperoxidase (MPO) and NF-*κ*B signaling pathway [84]. Echocardiography showed that left ventricular shortening fraction (LVFS) was significantly reduced and cardiac function was improved in IL-37-treated mice compared with the control group [84].

The above studies show that IL-37 may be a protective factor of atherosclerosis and ACS and may potentially serve as a new target for the diagnosis and treatment of these cardiovascular diseases (Table 7).

### 5.6. IL-37 in Obesity and Insulin Resistance

Obesity is currently considered to be a chronic inflammatory disease. Adipocytes in obese patients overexpress inflammatory factors, and chemokines, macrophages, and other immune cells are activated in this disease, leading to long-term, chronic inflammatory reactions [85]. Insulin resistance is closely related to the occurrence and development of chronic inflammation and abnormal expression of immune factors [86, 87]. Studies found that IL-37 may be closely related to obesity and insulin resistance (Table 8).

In a study included 21 obese patients, Moschen et al. [88] detected the changes in IL-1 family cytokines in liver and adipose tissue of patients who received laparoscopic adjustable gastric banding. They found that preoperative expression of IL-1 family cytokines was significantly higher in subcutaneous and visceral adipose tissue than in liver, whereas anti-inflammatory cytokines increased in adipose and liver tissues after bariatric surgery, and insulin resistance was improved [88]. Expression of IL-37 in the liver was positively correlated with body mass index but negatively correlated with *γ*-glutamyl transferase (GGT). Expression of IL-37 in subcutaneous adipose tissue was negatively correlated with BMI, serum insulin, and homeostasis model assessment (HOMA) index, and BMI, GGT, and HOMA index decreased after weight reduction.

Ballak et al. [89] found that expression levels of IL-37 mRNA in human adipose tissue were positively correlated with insulin sensitivity. Compared with wild-type mice, lower body weight gain, reduced number and size of adipocytes, decreased liver weight and hepatic triglyceride content, and reduced blood cholesterol levels were found in IL-37 transgenic mice after feeding with high-fat diet for 16 weeks. IL-37 inhibited the activation of multiple proinflammatory signaling pathways in adipose tissue and decreased the number of macrophages [89]. Recombinant IL-37 inhibited adipogenesis in adipocytes and activate adenosine 5′-monophosphate-activated protein kinase (AMPK) signaling pathway [89]. IL-37 can maintain the phosphorylation of IRS-1 tyrosine (Tyr) 941, so as to remedy downstream insulin signaling [89]. This indicates that IL-37 may play an important anti-inflammatory role in obesity-induced inflammation and insulin resistance in both mice and humans and may serve as a potential target for the treatment of obesity, insulin resistance, and type 2 diabetes.

## 6. Conclusion and Outlook

As a new member of IL-1 family, IL-37 is widely expressed in human organs and tissues. IL-37, as an inhibitor of inflammation, plays an important regulatory role in both innate immune response and adaptive immune response. IL-37 can inhibit the expression of a variety of inflammatory cytokines. Intracellularly, IL-37 forms complex with Smad3 to achieve nuclear translocation, which in turn regulates gene transcription, cell metabolism, cell proliferation, and cytokine expression and inhibits the activity of DCs. Extracellularly, IL-37 acts as an immunosuppressive agent by binding to IL-18R*α* to form complex with IL-18BP, so as to achieve the role of immune suppression by inhibiting the synthesis of IFN-*γ* and suppressing signal transduction after TLRs. Studies have confirmed that IL-37 plays an important role in many diseases such as infectious diseases, metabolic diseases, ischemia-reperfusion injury, autoimmune diseases, and tumors. In spite of those progresses achieved by previous studies, IL-37 receptors, related signaling pathways, mechanisms of signal transduction, and the interactions with other cytokines still haven't been completely elucidated. In addition, studies on IL-37 mainly focus on IL-37b, and functions of other isoforms are still unknown. In addition, most of the experiments were performed on cell lines and animal models, clinical studies still lack. In-depth study on the mechanism and key aspects of IL-37-mediated immune response may provide the theoretical basis for further clarifying the occurrence and development of immune response and further elucidating the pathogenesis of immune-related diseases and carcinomas. The identification of novel targets will definitely improve the treatment of these diseases.

## Figures and Tables

**Table 1:** Structures, molecular weights, functions, and expression of IL-37 isoforms.

Isoform	Structure	Molecular weight	Function	Expression	Reference
IL-37 All forms	Exons 1 to 6	17~25 kDa	Suppress immune responses Potential antitumor effect Not completely clear	Expressed in human, not detected in mice Yes in the following: Tissues: plasma cells in epithelial crypts and germinal centres of tonsils, lamina propria of colon, plasma cells of tonsil germinal centres and tonsil epithelial cells, skin sweat glands, skin sebaceous glands, colon epithelium, normal breast cells, placental syncytial trophoblast, skin epidermal cells, blood monocytes, keratinocytes in stratum granulosum of skin Organs: testis, thymus, uterus, muscle, brain, thalamus, lung, spleen, prostate, placenta; low levels in heart, adrenal glands, stomach, liver, salivary glands, pancreas, kidney Cell lines: PBMC, A431, THP-1, U937, IMTLH, KG-1, HL60, HPBMC, HPT-4, RAJI, SK-LU-1, CCL-247, NHDC Carcinomas: stroma of colon carcinomas, breast carcinomas, some colon carcinoma cells, melanomas, lung carcinomas, ductal mammary carcinoma, hepatocellular carcinoma No in the following: Prostate carcinoma cells, blood lymphocytes	[2–4, 9, 11–13, 21]

IL-37a Isoform 5	Exon 3 (prodomain) and exons 4 to 6	21.55 kDa	Anti-inflammatory effect Not completely clear	Yes in the following: Tissues and organs: lymph nodes, thymus, bone marrow, placenta, colon, lung, testis, placenta, colon, brain Cell lines: THP-1, U937, HL60, IMTLH, HPT-4 Carcinomas: colon carcinoma No in the following: Spleen, tonsil, foetal liver, liver, heart, skeletal muscle, kidney, pancreas, prostate, ovary, small intestine, leukocytes, T lymphocytes, B lymphocytes	[2, 4, 9, 11, 12]

IL-37b Isoform 1	Exons 1 and 2 (prodomain) and exons 4 to 6	24.13 kDa	Suppresses immune responses and inflammation Potential antitumor effect	Yes in the following: PBMC, lymph nodes, placenta, colon, lung, testis, kidney No in the following: brain, heart	[2–4, 11, 12]

IL-37c Isoform 4	Exons 1 and 2 (prodomain) followed by exons 5 and 6	19.61 kDa	May not have biological functions Downregulates IL-37b and IL-37d	Yes in the following: lymph nodes, placenta, colon, lung, testis, heart No in the following: brain, kidney	[2, 4]

IL-37d Isoform 2	Exon 1 and exons 4 to 6	21.95 kDa	Unknown	Yes in the following: testis, bone marrow No in the following: lymph nodes, placenta, colon, lung,brain, kidney, heart	[2, 4]

IL-37e Isoform 3	Exons 1, 5, and 6	17.46 kDa	May not have biological functions Downregulates IL-37b	Yes in the following: testis, bone marrow No in the following: lymph nodes, placenta, colon, lung, brain, kidney, heart	[2, 4]

PBMC: peripheral blood mononuclear cell. IMTLH: bone marrow stromal cell line. HPT-4: pancreas cell line.

**Table 2:** Functions, regulation, and mechanisms of IL-37.

Function, regulation, or mechanism	Reference
Suppresses innate immunity and inflammation	[3]
Suppresses adaptive immunity	[20]
Upregulated after stimulation of LPS, Pam3CSK4, and TGF-*β*1	[3]
Induced by IL-1*β*, TNF, IFN-*γ*, IL-18, TGF-*β*, and TLR ligands	[3]
Suppressed by GM-CSF combined with IL-4	[3]
Suppresses proinflammatory cytokines: IL-1*α*, IL-1*β*, IL-1Ra, IL-6, IL-8, IL-17, IL-23, TNF-*α*, and IFN-*γ*	[3, 8]
Suppresses chemokines: MIP-2/CXCL2, CCL12/MCP-5, and BCA-1/CXCL13	[3, 8]
Inhibits M-GSF and GM-CSF	[3, 8]
Increases the immunosuppressive factor TGF-*β*1	[3, 8]
Induces the expression of nitric oxide in vitro	[16]
Inhibits DCs functions	[20]
Attenuates T cell-mediated inflammation	[20]
Interacts with Smad3	[3]
Inhibits the STATs, p38MAPK, ERK1/2, JNK, FAK, Pyk2, paxillin, NF-*κ*B, kinase Fyn, TAK1	[3, 8, 12, 28–30]
Inhibits NLRP3 inflammasome	[3, 16]
Binds to IL-18R*α* to form a complex with IL-18BP, thereby reduces the activity of IL-18	[9, 16, 33, 36]
Binds to SIGIRR	[12, 16, 38]
Protective factor in mouse model of LPS-induced shock	[3]
Limits tissue injury during infections	[3, 16]
Potential protective factor in ischemia-reperfusion injury	[44, 49, 50]
Potential protective role in autoimmune diseases	[5, 6]
Potential antitumor effect	[7, 13, 21, 23–25]
Potential protective factor in cardiovascular diseases	[81, 84]
Related to obesity and insulin resistance	[88, 89]

TLR: Toll-like receptor. LPS: lipopolysaccharide. FAK: focal adhesion kinase. Pyk2: proline-rich tyrosine kinase 2; NLRP3: NOD-like receptor family Pyrin Domain-Containing 3. SIGIRR: single immunoglobulin IL-1 receptor related protein.

**Table 3:** Effects and mechanisms of IL-37 in infectious diseases.

Diseases	Model	Effect	Mechanism	Reference
Endotoxic shock	hIL-37tg mice	Attenuates shock state	↓IL-6 ↓IL-1*β* ↓IL-17 ↓IFN-*γ* ↓DCs	[3]
Aspergillus infection	Mice	Attenuates neutrophil infiltration, lung injury, and cystic fibrosis	↓NLRP3 inflammasome	[16]
Diffuse candidiasis	hIL-37tg mice	Increases mortality rate	↓TNF-*α* ↓Neutrophils recruitment	[42]

DCs: dendritic cells. ↓: downregulate.

**Table 4:** Protective effect and mechanisms of IL-37 in ischemia-reperfusion (I/R) injury.

Diseases	Model	Cell	Mechanism	Reference
Hepatic I/R injury	Mice	Hepatocytes and Kupffer cells	↓TNF-*α* ↓MIP-2 ↓ROS ↑Bcl-2	[44]
Myocardial I/R injury	Mice	Cardiomyocytes and neutrophils	↓ROS ↓TLR-4↓NF-*κ*B signaling ↓LIX ↓KC ↑IL-10 ↑Bcl-2/Bax ratio	[49]
Renal I/R injury	Mice	Tubular epithelial cells	↓TNF-*α* ↓IL-6 ↓IL-1*β*	[50]

TLR: Toll-like receptor. NF-*κ*B: nuclear factor kappa B. ROS: reactive oxygen species; ↑: upregulate. ↓: downregulate.

**Table 5:** Anti-inflammatory effects and mechanisms of IL-37 in autoimmune diseases.

Disease	Expression status	Cells	Model	Mechanism	Reference
Inflammatory bowel disease	^a^Colonic mucosa↑ ^b^Serum ↓	No	Colitis hIL-37tg mice	↑IL-10 ↓IL-1*β* ↓TNF-*α* ↓T cells ↓DCs	[53–56]
Systemic lupus erythematosus	^c^Serum/PBMC ↑	PBMCs	No	↓TNF-*α* ↓IL-6 ↓IL-1*β*	[58, 59]
Rheumatoid arthritis	^d^Synovial tissue/ plasma/PBMC ↑	PBMCs and naive CD4+ T cells	Collagen-induced arthritis mice	↓IL-17 ↓IL-1b ↓ IL-6 ↓Th17 cells	[61–64]
Psoriasis	^e^Psoriatic lesion↑	Keratinocyte cells	K14-VEGF-Tg mice	↓IFN-*γ* ↓CXCL8 ↓IL-6 ↓S100A7	[68]
Asthma	^f^PBMC/Serum/Induced sputum cells↓	Induced sputum cells	Ovalbumin-induced asthma mice	↓IL-1*β* ↓IL-6 ↓TNF-*α*↓IL-17	[70, 71]
Guillain-Barre syndrome	^g^Cerebrospinal fluid/Plasma↑	Cerebrospinal fluid and plasma cells	No	Unknown	[72]
Ankylosing spondylitis	^c^Serum/PBMC↑	PBMCs	No	↓TNF-*α* ↓IL-6 ↓IL-17 ↓IL-23	[73]
Graves' disease	^c^Serum/PBMC ↑	PBMCs	No	↓IL-6 ↓IL-17 ↓TNF-*α*	[74]

PBMC: peripheral blood mononuclear cell. DCs: dendritic cells. ↑: upregulate. ↓: downregulate. ^a^Levels of IL-37 in colonic mucosa were increased. ^b^Levels of IL-37 in serum were reduced. ^c^Expression of IL-37 in both serum and PBMC was increased. ^d^Expression of IL-37 in synovial tissue, plasma, and PBMC was elevated. ^e^Levels of IL-37 in psoriatic lesion were upregulated. ^f^Levels of IL-37 in PBMC, serum, and induced sputum cells were downregulated. ^g^Expression of IL-37 in both cerebrospinal fluid and plasma was increased.

**Table 6:** Antitumor effects and mechanisms of IL-37 in cancers.

Cancer type	Cell lines	Model	Mechanism	References
Fibrosarcoma	MCA205	Mice	Unclear, may be related to IFN-*γ*, functional T cells, and B cells	[13]
Hepatocellular carcinoma	Hep3B and Hepa1-6	Mice	↑CD57+ NK cells	[21]
Non-small-cell lung cancer	H1299	Mice	Unclear, may inhibit tumor angiogenesis, VGEF, and CD34	[23]
Renal cell carcinoma	BEL-7402	No	↓STAT3 pathway ↓IL-6 ↓HIF-1*α* ↓Bcl-2 ↓Cyclin D1	[24]
Cervical cancer	Hela and C33A	No	↓STAT3 pathway ↑TNF-*α*↑IL-1*β*	[25]
Oral squamous cell carcinoma	THP-1 and RAW264.7	No	↓IL-6 ↓TNF-*α*↓IL-1*β*	[76]

↑: upregulate. ↓: downregulate.

**Table 7:** Effects and mechanisms of IL-37 in cardiovascular disease.

Diseases	Model	Effect	Mechanism	Reference
Atherosclerosis	ApoE-deficient diabetic mice	Attenuate vascular calcification and atherosclerosis	↑OPG ↓ALP ↓BMP-2 ↓TNF-*α* ↓IL-18 ↑IL-10	[81]
Acute myocardial infarction	Mice of acute myocardial infarction	Improve myocardial infarction mice cardiac function	↓MPO ↓NF-*κ*B signaling	[84]

OPG: osteoprotegerin. ↑: upregulate. ↓: downregulate.

**Table 8:** IL-37 in obesity and insulin resistance.

Disease	Expression status	Model	Mechanism	Reference
Obesity	^a^Liver and subcutaneous adipose tissue	No	Unknown	[88]
Insulin resistance	^b^Adipose tissue	High-fat diet mice	↓ERK1/2 ↓JNK ↓IKK*α*/*β* ↓macrophage ↓NK cells ↓cytotoxic T cells ↓IL-1*β* ↓IL-6 ↓CXCL1 ↓TLRs signaling ↑phosphorylation of IRS-1 tyrosine 941 ↑AMPK signaling	[89]

TLR: Toll-like receptor. ↑: upregulate. ↓: downregulate; ^a^IL-37 expression was increased after weight loss in liver and subcutaneous adipose tissue, ^b^IL-37 expression level was higher in adipose tissue compared with stromal vascular cells and relates to insulin sensitivity.
